# Transforming growth factor beta receptor type III is a tumor promoter in mesenchymal-stem like triple negative breast cancer

**DOI:** 10.1186/bcr3684

**Published:** 2014-07-01

**Authors:** Bojana Jovanović, J Scott Beeler, Michael W Pickup, Anna Chytil, Agnieszka E Gorska, William J Ashby, Brian D Lehmann, Andries Zijlstra, Jennifer A Pietenpol, Harold L Moses

**Affiliations:** 1 Department of Cancer Biology, Vanderbilt-Ingram Cancer Center, Vanderbilt University School of Medicine, 2220 Pierce Avenue, 698 Preston Research Building, Nashville, TN 37232, USA; 2 Department of Biochemistry, Vanderbilt University School of Medicine, 23rd and Pierce, 607 Light Hall, Nashville, TN 37232, USA; 3 Scibrary the Science Library, McDonough, GA 30253, USA; 4 Department of Pathology, Microbiology, and Immunology, Vanderbilt University School of Medicine, 1161 21st Avenue South, C-3321A Medical Center North, Nashville, TN 37232, USA

## Abstract

**Introduction:**

There is a major need to better understand the molecular basis of triple negative breast cancer (TNBC) in order to develop effective therapeutic strategies. Using gene expression data from 587 TNBC patients we previously identified six subtypes of the disease, among which a mesenchymal-stem like (MSL) subtype. The MSL subtype has significantly higher expression of the transforming growth factor beta (TGF-β) pathway-associated genes relative to other subtypes, including the TGF-β receptor type III (TβRIII). We hypothesize that TβRIII is tumor promoter in mesenchymal-stem like TNBC cells.

**Methods:**

Representative MSL cell lines SUM159, MDA-MB-231 and MDA-MB-157 were used to study the roles of TβRIII in the MSL subtype. We stably expressed short hairpin RNAs specific to TβRIII (TβRIII-KD). These cells were then used for xenograft tumor studies *in vivo*; and migration, invasion, proliferation and three dimensional culture studies *in vitro*. Furthermore, we utilized human gene expression datasets to examine TβRIII expression patterns across all TNBC subtypes.

**Results:**

TβRIII was the most differentially expressed TGF-β signaling gene in the MSL subtype. Silencing TβRIII expression in MSL cell lines significantly decreased cell motility and invasion. In addition, when TβRIII-KD cells were grown in a three dimensional (3D) culture system or nude mice, there was a loss of invasive protrusions and a significant decrease in xenograft tumor growth, respectively. In pursuit of the mechanistic underpinnings for the observed TβRIII-dependent phenotypes, we discovered that integrin-α2 was expressed at higher level in MSL cells after TβRIII-KD. Stable knockdown of integrin-α2 in TβRIII-KD MSL cells rescued the ability of the MSL cells to migrate and invade at the same level as MSL control cells.

**Conclusions:**

We have found that TβRIII is required for migration and invasion *in vitro* and xenograft growth *in vivo*. We also show that TβRIII-KD elevates expression of integrin-α2, which is required for the reduced migration and invasion, as determined by siRNA knockdown studies of both TβRIII and integrin-α2. Overall, our results indicate a potential mechanism in which TβRIII modulates integrin-α2 expression to effect MSL cell migration, invasion, and tumorigenicity.

## Introduction

The term ‘triple negative breast cancer’ (TNBC) is used to classify the 10% to 20% of all breast cancers that lack estrogen receptor (ER) and progesterone receptor (PR) expression as well as amplification of the human epidermal growth factor receptor 2 (HER2) [1]. Disease heterogeneity and the absence of well-defined molecular targets have made treatment of TNBC challenging. There is a major need to understand better the molecular basis of this type of breast cancer in order to develop effective therapeutic strategies [1]. In a previous study, we performed gene expression (GE) analyses and identified six distinct molecular TNBC subtypes with unique biological drivers [2], including one that was enriched for mesenchymal-associated genes termed mesenchymal-stem like (MSL). The MSL subtype is characterized by increased expression of genes related to transforming growth factor beta (TGF-β) signaling as well as pathways that play roles in extracellular matrix (ECM), focal adhesion, cell motility and cell differentiation [2]. Of note, TGF-β receptor type III (TβRIII) (gene symbol: *TGFBR3*) was among the differentially expressed TGF-β signaling components in the MSL subtype.

The TGF-β signaling pathway has been implicated in cancer initiation and progression through tumor cell autonomous and non-autonomous signaling [3,4]. Initially identified as a tumor suppressor and then as a mediator of tumor progression, TGF-β signaling demonstrates diverse capabilities in cancer. The TGF-β pathway suppresses tumor growth through regulation of epithelial and stromal cell signaling [5]. Dysfunction of the pathway leads to carcinoma progression and metastasis [3]. While there has been significant focus on TGF-β receptor type I (TβRI) and TGF-β receptor type II (TβRII), research on TβRIII has lagged. Prior studies have demonstrated that TβRIII can regulate TGF-β signaling either via delivering TGF-β2 ligand to TβRII [6-9] or by binding to the cytoplasmic domain of TβRII, forming an active TβRI-TβRII signaling complex [10-13]. Currently, analysis of gene expression data sets generated from multiple cancer types indicates that TβRIII expression is decreased in higher-grade cancers [14-17]. However, the role of TβRIII is controversial in breast cancer, since it has been reported that TβRIII can act as either a tumor suppressor or promoter in this cancer [18,19].

In the current study, we focused our investigations on the functional role of TβRIII in the MSL subtype of TNBC. We used a loss-of-function approach in representative MSL cell lines to demonstrate that TβRIII is required for maintenance of tumorigenicity in MSL TNBC cell lines and that regulation of integrin-α2 (gene symbol: *ITGA2*) expression is mechanistically involved in the observed phenotypes. This study demonstrates that TβRIII promotes the *in vivo* growth of a subset of TNBC and provides a pre-clinical rationale for consideration of TβRIII as a potential target for further discovery efforts.

## Materials and Methods

### Cell culture

SUM159 cells (Asterand, Detroit, MI, USA) were maintained in (Dulbecco’s) Modified Eagle’s Medium: Nutrient Mixture F12 ((D)MEM-F12, GIBCO, Grand Island, NY, USA) supplemented with 5% fetal bovine serum (FBS) (GIBCO) and 0.5 μg/ml hydrocortisone. MDA-MB-231 and MDA-MB-157 (ATTC, Manassas, VA, USA) were maintained in (D)MEM (GIBCO) supplemented with 10% FBS. Stable TβRIII-KD SUM159 cell lines were generated by lentiviral infection with virus carrying four independent short hairpin RNA (shRNA) clones (sequence-verified shRNA, pLKO.1-puro), (Sigma-Aldrich, St. Louis, MO, USA), Mission shRNA library #SHCLNG-NM_003243: clone#TRCN0000033433 (TβRIII-KD), clone#TRCN0000359000 (TβRIII-KD2), clone#TRCN0000359001 (TβRIII-KD3), and clone# TRCN0000359081 (TβRIII-KD4)) followed by puromycin selection (Invitrogen-Life Technology, Inc, Carlsbad, CA, USA). MDA-MB-231 and MDA-MB-157 were stably infected with clone# TRCN0000033433. Integrin-α2 was stably knocked down in TβRIII-KD MSL cells using lentiviral particles carrying shRNA to integrin-α2 (α2-KD) (Sigma-Aldrich, Mission shRNA validated library, #SHCLNG-NM_002203, clone#TRCN0000308081).

### Three-dimensional culture assay

The wells in 48-well plates were coated with 50 μl of growth factor reduced BD Matrigel (BD Biosciences #356231, San Jose, CA, USA) and allowed to polymerize at 37°C for 15 minutes. Then, 5 x 10^5^ cells were resuspended in 200 μl of growth factor reduced BD Matrigel and plated onto the matigel-coated wells. Plates were incubated for 30 minutes after which 1 ml of media was added to the top of the matrigel. Media was replenished every 48 hours. Images were taken at day six. Quantification of the images was performed using Fiji Software.

### Cell proliferation assays

#### Cell counts

Cells were plated into six-well plates at a density of 1.25 x 10^5^ cells/well. The following day cells were treated with 1 ng/ml TGF-β1 (R&D Systems, #102-B1, Minneapolis, MN, USA) and TGF-β2 (R&D Systems, #102-B2). After 72 hours treatment with TGF-β, viable cells were counted.

#### ^3^H-Thymidine incorporation assay

A total of 2.5 × 10^4^ cells were plated in a 24-well dish and allowed to grow overnight. The next day the medium was aspirated and replaced with complete medium containing +/−TGF-β1 or TGF-β2 (1 ng/ml). The cells were then subjected to [^3^H] thymidine incorporation assay as previously described [20].

### Migration and invasion assays

#### Magnetic attachable stencils migration assays

This migration method serves as a more reproducible alternative to the scratch assay. The use of magnetic force to attach stencil to the multi-well plates is a new strategy that creates defined and reproducible cell-free voids for quantitation of cell migration and has been well characterized and described by Ashby *et al*. [21]. Magnetic attachable stencils (MAtS) were attached to the surfaces of each well of a 12-well plate by placing a platform with magnets underneath and in direct contact with the 12-well plate. Cells were then plated in triplicate at 7.5 x 10^5^ cells per well around the MAtS in serum-free media. The next day the MAtS were removed and cells were treated with 1 ng/ml TGF-β1 (R&D Systems, #102-B1) and 1 ng/ml TGF-β2 (R&D Systems, #102-B2). Gap closure was quantified (Tscratch software) at both 0 and 24 hours and percent of closure determined with the following equation: percent of closure = average of ((gap area: 0 hour) – (gap area: 24 hours))/(gap area: 0 hour) using images from 12 different microscopic fields per well (4X magnification).

#### Transwell assays

Migrations (Costar, #3422, Tewksbury, MA, USA) were conducted by plating 2.5 x 10^4^ cells in the top of the transwell and media with 10% FBS in the bottom of the well to act as a chemoattractant. Cells were fixed in 4% paraformaldehyde and stained with 4′, 6-diamidino-2-phenylindole (DAPI). Quantification was performed by taking pictures of multiple regions of the membrane after which cells’ nuclei were counted using Metamorph software. The same migration assay was used to measure blocked integrin-α2 function. The TβRIII-KD cells were incubated for 30 minutes with integrin-α2 blocking antibody (Abcam, #ab24697, Cambridge, MA, USA) washed two times with PBS and plated in the top of the transwell. Invasion assays were conducted by plating 5 x 10^5^ cells in serum-free media in the upper chamber, pre-coated with growth factor reduced matrigel. In the bottom chamber (D)MEM with 10% FBS was used as a chemoattractant (BD Biosciences, #354483). Cells that had invaded through the matrigel were fixed in 4% paraformaldehyde and stained using DAPI. Quantification of cells that invaded into the matrigel was performed using the same protocol as described for the transwell assays.

### Xenograft tumor studies

One milllion cells embeded in collagen were implanted into the number four gland of six- to eight-week-old female athymic nude- Foxn1^nu/nu^ mice (purchased from Harlan Sprague- Dawley, Inc., Indianapolis, IN, USA). Mice were monitored weekly for tumor growth. Tumor measurements were performed once a week for five weeks after palpable tumors formed. Tumor volume was measured at the indicated times with calipers, and tumor volumes were calculated as width^2^ x length/2. All mouse experiments were approved by the Vanderbilt University Institutional Animal Care and Use Committee (IACUC).

### Luciferase reporter assay

Cells were seeded at a density of 2 X 10^4^ cells/well in 12-well tissue culture plates. The following day, the cells were transiently transfected using Transfectin lipid reagent following the manufacturer’s protocol (Bio-Rad #170-3351, Hercules, CA, USA). Cells were transfected with 1.5 μg 3TP-Lux [22] or CAGA(9)-Luc [23]. pRL-CMV-renilla (Promega #E2261, Madison, WI, USA) was co-transfected and used as an internal control to correct for transfection efficiency. Eighteen hours after transfection, cells were treated with 1 ng/ml TGF-β1 or TGFβ-2 (R&D Systems, #102-B1 and #102-B2, respectively). Twenty-four hours after TGF-β treatment, cells were harvested and assayed for promoter specific luciferase activity using a Dual-Luciferase Reporter Assay System (Promega #E1910) according to the manufacturer’s protocol. Luciferase activity was measured using a BD/Pharmigen Monolight 3010 luminometer.

### RNA preparation and quantitative PCR

RNA was isolated and purified using an RNeasy Mini Kit and an RNase-Free DNase Set (Qiagen, Valencia, CA, USA). A total of 750 μg of RNA was used to synthesize cDNA using Superscript III reverse transcriptase as described by the manufacturer (Invitrogen). Bio-Rad iCycler and CFX96 machines were used for qPCR employing *Power* SYBR Green (Applied Biosystems, Carlsbad, CA, USA) or SsoAdvanced SYBR Green Supermix (Bio-Rad), respectively. *C*_*t*_ values were normalized to GAPDH for statistical analyses. Primer sequences are available in Additional file 1.

### Immunoblotting

Standard protein preparation and electrophoresis procedures were used as described [4]. Western membranes were blocked in 5% milk and incubated with primary antibody overnight. The antibody list with concentrations and the catalog numbers are available in Additional file 1.

### Flow cytometry

Cells were detached using Accutase (Life Technologies), pelleted, washed and counted. One million cells were incubated with TβRIII antibody (Cell Signaling, #5544, Danvers, MA, USA) for 30 minutes, washed, and then incubated at 4°C with Alexa Fluor 488 conjugated secondary antibody (Life Techologies, #A11034) for 30 minutes. One million cells were labeled with fluorescence-conjugated integrin-α2 antibody (BioLegend, #314308, San Diego, CA, USA) for 30 minutes at 4°C. Cells were washed three times then analyzed on a FACSCalibur flow cytometer (Becton Dickinson, San Jose, CA, USA) using CellQuest Pro software. Data were analyzed with FlowJo software (Tree Star).

### Microarray gene expression analysis

#### Public database analysis

Human tissue and cell line microarray datasets were analyzed using GeneSpring GX 12.0 microarray analysis software (Agilent). Previously published TNBC gene expression profiles (n = 587 patients) [2] consisting of publicly available microarray data sets (the GEO registration numbers are referenced in Additional file 1) were obtained and processed as previously described and were in compliance with ethical requirements [2]. Comparisons between expression of *TGFBR3* and *ITGA2* for different TNBC subtypes were performed in R 3.0.1 [24] using the *t test* function for paired two-tailed Student’s *t*-tests and graphically represented using ggplot2 [25].

#### In vitro *three*-*dimensional culture analysis*

vRNA was extracted from SUM159 three-dimensional culture samples and hybridized to the human gene 1.0ST array, scanned with Affymetrix using AGCC v. 3.2.4 and then analyzed in R 3.0.1 using the oligo package. Samples were normalized with the RMA algorithm, genes were annotated with the pd.hugene.1.0.st.v1 package, and differential gene expression analysis was conducted using the limma package. The three-dimensional culture microarray data discussed in this publication have been deposited in the National Center for Biotechnology Information (NCBI)’s Gene Expression Omnibus [26] and are accessible through GEO Series accession number GSE54756 [27].

### Statistical analysis

All data were analyzed using the unpaired two-tailed Student’s *t* test (GraphPad Prism 5). Error bars show mean ± SEM. A two-sided *P* value less than 0.05 was considered significantly different.

## Results

### Human mesenchymal stem-like triple negative breast tumors and representative cell lines have increased TβRIII expression

**Figure 1:**
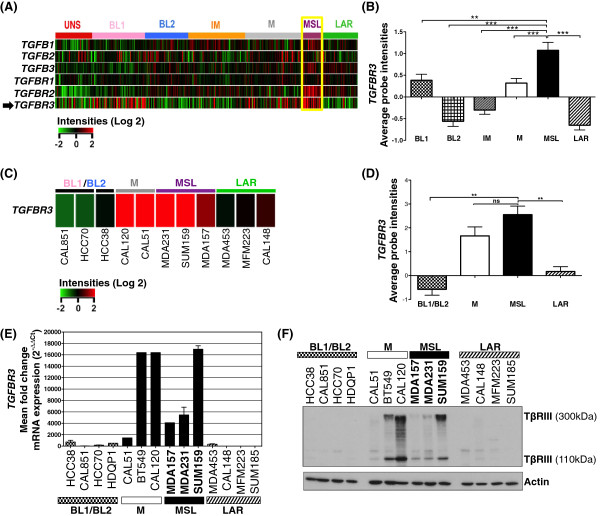
***TGFBR3 gene *expression levels are elevated in the mesenchymal stem-like (MSL) subtype of TNBC. A)** Heat map representation of gene expression for 587 TNBC tumors for each TGF-β ligand and receptor. **B)** Quantification of average *TGFBR3* gene expression across TNBC tumor subtypes, average based on individual TNBC tumor probe intensity values (**P = 0.004; ***P <0.0003 for a two-tailed Student’s *t*-test). **C)** Heat map of *TGFBR3* mRNA expression in TNBC representative cell lines. **D)** Quantification of *TGFBR3* gene expression across representative TNBC cell lines (**P = 0.004; for a two-tailed Student’s *t*-test, ns = not significant). **E)** qRT-PCR analysis of *TGFBR3* average mRNA expression (2^-ΔΔCt^) [28] from representative TNBC cell lines; graph bars represent the mean of three replicates with SEM error bars. **F)** Immunoblot analysis of TβRIII protein expression in TNBC representative cell lines, results representative of two independent experiments. SEM, standard error of the mean; TNBC, triple negative breast cancer; TβRIII, type III transforming growth factor-beta receptor.

Using a gene expression data set generated from 587 TNBC tumors, we examined the relative mRNA levels of TGF-β receptors and ligands across subtypes of TNBC. We observed elevated expression of *TGFBR3* in basal-like1 (BL1), mesenchymal (M) and MSL tumors (Figure 1A). The highest relative level of *TGFBR3* expression was in the MSL subtype (Figure 1B). Average probe intensities for the TGF-β receptors I and II as well as TGF β ligands 1 and 3 were also elevated in the MSL subtype in comparison to the rest of the TNBC subtypes (Additional file 2: Figure S1). Similarly, analysis of *TGFBR3* gene expression across a panel of TNBC cell lines, representative of the various subtypes, demonstrates that the M and MSL subtypes have relatively higher levels of *TGFBR3* mRNA (Figure 1C-D). These findings were validated by qPCR (Figure 1E) and immunoblot analyses for TβRIII protein levels (Figure 1F). Although the TNBC M subtype cell lines also showed increased levels of TβRIII expression, we focused our studies of this receptor on the MSL subtype as their expression is more consistent with human datasets (Figure 1A-B).

### Knockdown of TβRIII in MSL TNBC cells leads to decreased tumorigenicity *in vivo*

**Figure 2:**
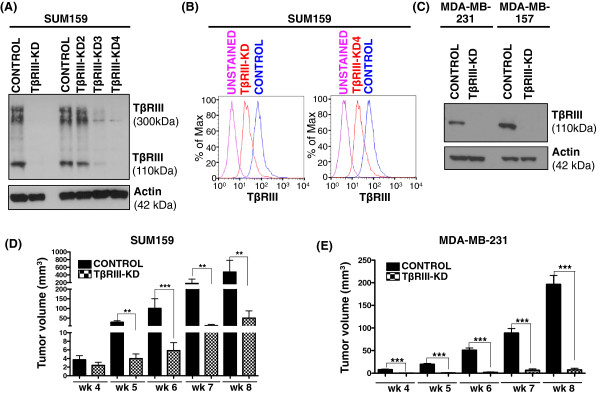
**Stable knockdown of TβRIII in MSL TNBC cells reduces xenograft tumor growth*. *A)** Immunoblot analysis of TβRIII protein expression in lysates harvested from SUM159 cells stably expressing control and four different TβRIII shRNA vectors (TβRIII-KD, KD2-4). **B)** Flow cytometry analysis of TβRIII protein levels in SUM159 controls, TβRIII-KD and TβRIII-KD4. **C)** Immunoblot analysis of TβRIII protein expression in lysates harvested from MDA-MB-231 and MDA-MB-157 cells stably expressing control and TβRIII-KD. **D-E)** Tumors in nude mice were palpable three weeks post implantation of the MSL cell line (D, SUM159 and E, MDA-MB-231). Serial tumor volumes (mm^3^) were measured weekly for five weeks post palpation for both controls and TβRIII-KD. Each data bar represents the mean tumor volume of 10 tumors; error bar represents SEM (**P ≤0.005, ***P <0.0001 for a two-tailed Student’s *t*-test). MSL, mesenchymal stem-like; SEM, standard error of the mean; TNBC, triple negative breast cancer; TβRIII, type III transforming growth factor-beta receptor.

In order to determine the significance of the TβRIII expression in MSL TNBC cell behavior, we knocked down TβRIII in MSL cells and performed orthotopic xenograft tumor studies. We used a panel of four shRNA expression vectors to optimize TβRIII knockdown, as validated by immunoblot and flow cytometry analyses (Figure 2A-C). We utilized immunocompromised nude mice to establish orthotopic xenograft tumors from cell lines representing the MSL subtype of TNBC with and without TβRIII knockdown. Initially we tested SUM159 cells with two shRNA expression vectors (TβRIII-KD and TβRIII-KD4) to eliminate off target effects of the shRNA (Additional file 2: Figure S2). After establishing that both expression vectors resulted in a similar phenotype, we used a single shRNA (TβRIII-KD) in all subsequent experiments across three MSL cell lines. Knockdown of TβRIII in the SUM159 and MDA-MB-231 MSL cell lines significantly decreased xenograft tumor growth (Figure 2D-E). MDA-MB-157 showed inconsistent results (Additional file 2: Figure S3A) and after further investigation we discovered that the TβRIII-KD tumors expressed TβRIII (Additional file 2: Figure S3B). Thus, either there was a selection against the knockdown *in vivo* and, therefore, the tumor cells expressed TβRIII, or there was a small subpopulation of MDA-MB-157 cells at the start of the experiment that retained expression and seeded the tumor growth. Regardless, both explanations provide further evidence for the tumor-promoting effect of TβRIII.

### Knockdown of TβRIII in MSL cell lines does not affect cell proliferation or viability

**Figure 3:**
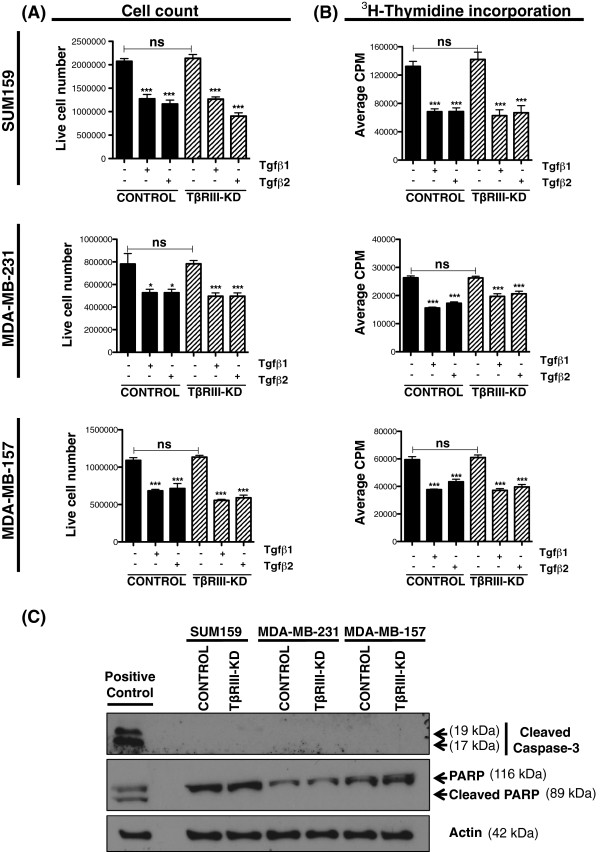
**TβRIII-KD in MSL cell lines does not affect cell proliferation. A)** Live cell count proliferation assay for SUM159, MDA-MB-231 and MDA-MB-157 controls versus TβRIII-KD 72 hours post treatment with TGF-β1 and TGF-β2 ligands; graph bars represent the mean of six replicates with SEM error bars (ns = not significant; *P <0.01, ***P ≤0.0005 for a two-tailed Student’s *t*-test). **B)** Thymidine incorporation proliferation assay for SUM159, MDA-MB-231 and MDA-MB-157 controls versus TβRIII-KD in the presence or absence of TGF-β1 and TGF-β2 ligands; graph bars represent the mean of six replicates with SEM error bars (***P ≤0.0004 for a two-tailed Student’s *t*-test). **C)** Immunoblot analysis of cleaved-caspase 3 and PARP protein expression using lysates harvested from SUM159, MDA-MB-231 and MDA-MB-157 stably expressing control and TβRIII-KD. Results are representative of three independent experiments. MSL, mesenchymal stem-like; SEM, standard error of the mean; TGF-β, transforming growth factor beta; TβRIII-KD, type III transforming growth factor-beta receptor knockdown.

Since TβRIII-KD markedly decreased the tumorigenic potential of mesenchymal TNBC cells, we further explored whether this was due to a proliferation defect. TβRIII can bind to all TGF-β ligands but with highest affinity for TGF-β2 [29,30]; therefore, cells were treated with TGF-β2 in addition to TGF-β1. Both controls and TβRIII-KD MSL cell lines responded similarly to the ligands (Figure 3A-B). TβRIII-KD did not alter the proliferation rates of MSL cell lines (SUM159, MDA-MB-231 and MDA-MB-157) by live cell counts (Figure 3A) or ^3^H-thymidine incorporation assay (Figure 3B). Consistent with an intact TGF-β signaling pathway [22,23] we have observed an increase in phospho-SMAD2 following ligand treatment (Additional file 2: Figure S4). In order to examine cell viability and determine whether knockdown of TβRIII influenced apoptosis, we analyzed cleaved-caspase 3 and cleaved-PARP and we did not detect any difference between control and TβRIII-KD MSL cells (Figure 3C).

### Knockdown of TβRIII in MSL cells impairs motility, invasion and the ability to form invasive protrusions in three-dimensional cultures

**Figure 4:**
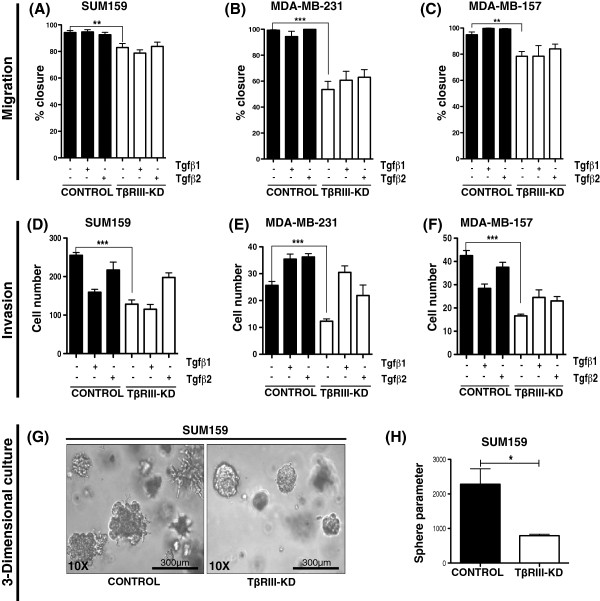
**Phenotypic effect of TβRIII knockdown in MSL cells. A-C)** Cells were plated around magnetic stencils. After cells had adhered the magnetic stencils were removed and migration assay was monitored for 24 hours. Bar graphs represent percentages of closure for each MSL cell line with TβRIII-KD in comparison to control with or without TGF-β1 and TGF-β2 treatment; graph bars represent the mean of three replicates with SEM error bars (**P = 0.001, ***P <0.0001 for a two-tailed Student’s *t*-test). **D-F)** Number of MSL cells that invaded through matrigel pre-coated transwells with or without 24 hour pre-treatment of cells with TGF-β1 and TGF-β2 ligands (***P <0.0001 for a two-tailed Student’s *t*-test). **G)** Representative 10x images of SUM159 controls versus TβRIII-KD cells embedded in three-dimensional matrigel culture. Scale bar: 300 μm. **H)** Quantification of SUM159 three-dimensional matrigel culture; bar graph represent tumor-sphere perimeter derived from mean of three replicates with SEM error bars (*P = 0.029 for a two-tailed Student’s *t*-test). MSL, mesenchymal stem-like; SEM, standard error of the mean; TGF-β, transforming growth factor beta; TβRIII-KD, type III transforming growth factor-beta receptor knockdown.

Using a validated method (please see methods section for details) for measurement of cell migration [21], we found that TβRIII-KD significantly decreased the migration of SUM159, MDA-MB-231 and MDA-MB-157 cells (Figure 4A-C). Treatment with TGF-β ligands had no effect on migration. In order to determine the invasive properties of MSL lines we analyzed their ability to migrate through a barrier using an invasion transwell assay. TβRIII-KD impaired the ability of the MSL cell lines to invade through matrigel pre-coated transwells and the addition of TGF-β ligands had little effect on invasion in either controls or knockdowns (Figure 4D-F). Next, we examined the effect of TβRIII-KD on the ability of MSL cells to form colonies in three-dimensional matrigel culture. After five days in culture, SUM159 cells with TβRIII-KD had smooth edges around cell spheres while control cells had multiple protrusions invading into the surrounding matrix (Figure 4G). These results were quantified by calculations of the perimeter, which show a significant difference between controls and TβRIII-KD (Figure 4H). Overall, these data indicate that TβRIII modulates migration and invasion, independent of TGF-β stimulation, in MSL cells. To further investigate TGF-β pathway signaling [31] in the MSL lines we used standard CAGA-luc (Additional file 2: Figure S5A) and 3TP-lux (Additional file 2: Figure S5C) reporter assays for measurement of TGF-β activity [22,23]. Assays were performed either in the presence of TGF-β1 or TGF-β2 ligands and compared to untreated controls [30,32]. In addition, we performed qPCR analysis for SMAD7 (Additional file 2: Figure S5B) and PAI-1 (Additional file 2: Figure S5D) gene expression as readout for downstream targets for canonical and non-canonical TGF-β activity, respectively [33,34]. The results of both assays indicate that knockdown of TβRIII does not modulate either arm of the TGF-β signaling pathway. Thus, MSL lines with TβRIII knockdown have resulting phenotypic changes without concomitant changes in the TGF-β signaling pathways measured. Considering these results and knowing that TβRIII can also bind to bone morphogenetic proteins (BMPs) [35], we treated the engineered MSL cell lines with BMP4. We did not observe significant changes in Smad1/5/8 phosphorylation in TβRIII-KD versus control MSL cells (data not shown). The results suggest that TβRIII modulates the tumorigenic potential of MSL TNBC cells through other signaling pathways.

### Knockdown of TβRIII is associated with increased expression of integrin-α2 in MSL TNBC cells

**Figure 5:**
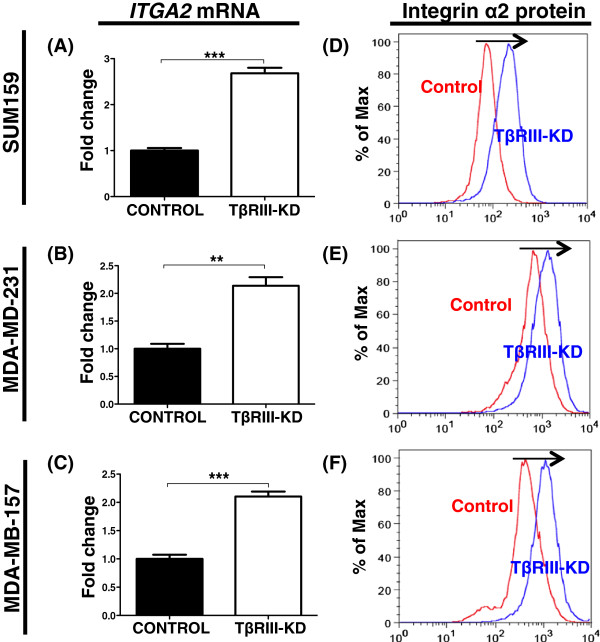
**TβRIII-KD modulates expression of *ITGA2 *in MSL cells. A-C)** qRT-PCR analysis for *ITGA2* mRNA expression from MSL TNBC cell lines with TβRIII-KD; graph bars represent the mean of three replicates with SEM error bars (**P = 0.003, ***P ≤0.0007 for a two-tailed Student’s *t*-test)*.***D-F)** Flow cytometry analysis for integrin-α2 in controls and TβRIII-KD MSL; arrow pointing to the right shows a shift towards an increase in protein levels of integrin-α2 in TβRIII-KD MSL cell lines. MSL, mesenchymal stem-like; SEM, standard error of the mean; TβRIII-KD, type III transforming growth factor-beta receptor knockdown.

To determine which genes and/or signaling pathways are significantly altered in MSL cells after TβRIII knockdown, we performed gene expression microarray analyses on SUM159 cells grown in three-dimensional cultures. The integrin signaling pathway, along with other cell adhesion pathways, were among the most significant pathways differentially expressed in TβRIII-KD MSL cells relative to control cultures (Additional file 3: Table S1). Analysis of individual genes of the integrin pathway revealed that *ITGA2* was a top gene that was significantly increased upon TβRIII knockdown (Additional file 3: Table S2). *In vitro* qRT-PCR analysis indicates a statistically significant (above two-fold) upregulation of integrin-α2 in the TβRIII-KD MSL cells (Figure 5A-C). The upregulation of integrin-α2 was further validated by flow analysis across all MSL (Figure 5D-F).

### TβRIII modulation of integrin-α2 expression is required for the migratory and invasive MSL cell line phenotypes

**Figure 6:**
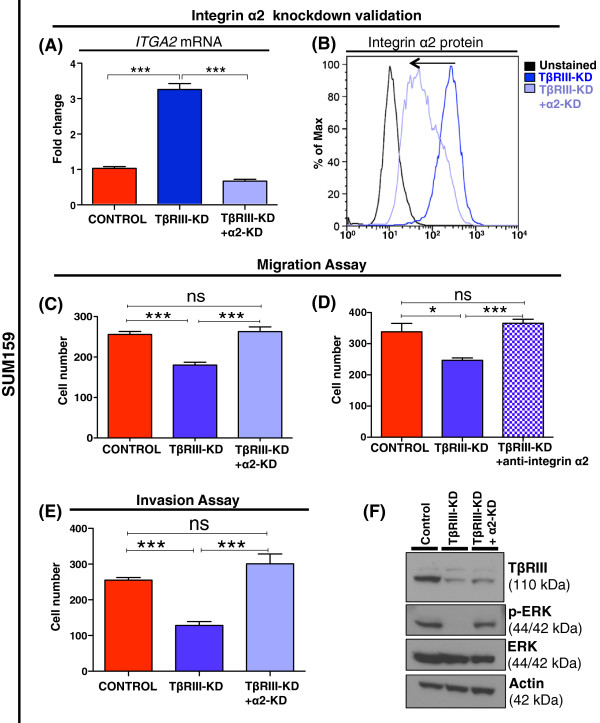
**Knockdown of integrin-α2 in TβRIII-KD MSL cells reverses migratory and invasive TβRIII-KD phenotypes. A)** qRT-PCR analysis for *ITGA2* mRNA expression before and after stable knockdown of integrin-α2 (α2-KD) in SUM159 cells with TβRIII-KD; graph bars represent the mean of the replicates with SEM error bars (***P <0.0001 for a two-tailed Student’s *t*-test). **B)** Flow cytometry analysis of α2-KD; arrow pointing to the left shows a shift towards a decrease in protein amount of integrin-α2 in TβRIII-KD SUM159 cell lines after α2-KD. **C)** Transwell migration assay representing number of cells migrated through transwell upon α2-KD in TβRIII-KD SUM159 cell line (ns = not significant, ***P <0.0001 for a two-tailed Student’s *t*-test); bar graph represents a mean of three replicates with SEM error bars. **D)** Transwell migration assay representing number of cells migrated upon treatment of TβRIII-KD SUM159 cell line with anti-α2 blocking antibody (ns = not significant, *P = 0.011, ***P <0.0001 for a two-tailed Student’s *t*-test). **E)** Transwell invasion assays with inserts pre-coated with matrigel allowing for testing the number of cells that can invade upon α2-KD in TβRIII-KD SUM159 cells (ns = not significant, ***P <0.0001 for a two-tailed Student’s *t*-test). **F)** Immunoblot analysis for phospho-ERK using protein harvested from SUM159 cells with TβRIII-KD and TβRIII-KD /α2-KD. MSL, mesenchymal stem-like; SEM, standard error of the mean; TβRIII-KD, type III transforming growth factor-beta receptor knockdown.

Using a clinically relevant, spontaneous mouse model of breast cancer progression and metastasis, Ramirez *et al*. demonstrated that integrin-α2β1 acts as a tumor suppressor and α2-null cells were more motile and invasive [36]. The *in vivo* and *in vitro* findings were further correlated with analysis of microarray gene expression datasets of human breast and prostate cancers, which showed a correlation between decreased expression of *ITGA2* and poor prognosis. Considering this role of integrin-α2 in breast cancer, we hypothesized that the decrease in migration and invasion upon TβRIII-KD in MSL cells could be rescued by concomitant knockdown of integrin-α2. To test our hypothesis, we stably knocked down integrin-α2 (α2-KD) in the MSL TβRIII-KD cells and performed migration and invasion assays (Figure 6A-B and Additional file 2: Figure S6A-B). Knockdown of integrin-α2 was sufficient to reverse the migration (Figure 6C and Additional file 2: Figure S6C) and invasion (Figure 6E and Additional file 2: Figure S6D) phenotype of MSL cells with TβRIII-KD to those of control cells. In addition, using an integrin-α2 neutralizing antibody we rescued the migratory phenotype (Figure 6D) in a manner similar to that observed after α2-KD in TβRIII-KD cells. Knelson and colleagues showed that knockdown of TβRIII leads to diminished fibroblast growth factor 2 (FGF2)-mediated ERK phosphorylation [37]. Consistent with this previous study, after knockdown of TβRIII in the MSL cells, the phospho-ERK levels decreased and were restored in the cells after simultaneous integrin-α2 and TβRIII knockdown (Figure 6F and Additional file 2: Figure S6E).

### Relationship between gene expression of TβRIII and integrin-α2 in TNBC patient dataset

**Figure 7:**
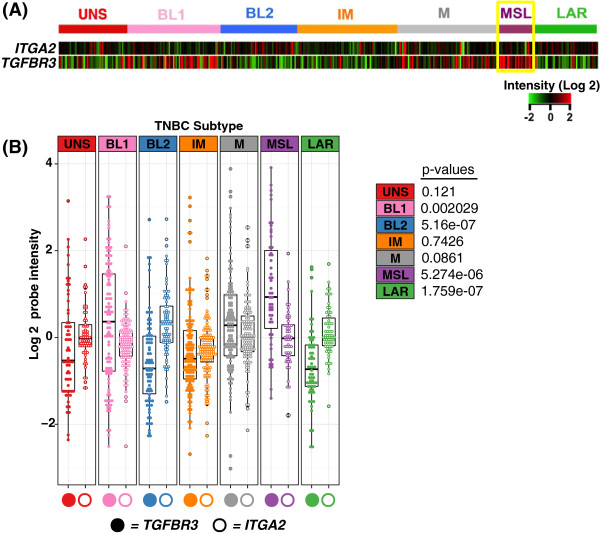
**TNBC patient dataset shows that expression of *TGFBR3 *is inversely correlated with expression of *ITGA2*. A)** Heat map representation of *TGFBR3* and *ITGA2* gene expression for 587 TNBC tumors for each *TGFBR3* and *ITGA2*. **B)** Quantification of gene expression for *TGFBR3* (solid circles) and *ITGA2* (empty circles) across TNBC tumor subtypes using log2 probe intensity values. The whiskers of the boxplot extend to the highest value that is within 1.5 interquartile range*. P* values were generated by performing a paired two-tailed Student’s *t*-test. TNBC, triple negative breast cancer.

To further investigate the association between TβRIII (*TGFBR3)* and integrin-α2 (*ITGA2*) in TNBC, we used the TNBC patient dataset described in Figure 1A [2] to analyze the relationship between *TGFBR3* and *ITGA2* gene expression. Our results indicate an inverse correlation between *ITGA2* and *TGFBR3* expression across TNBC subtypes. In particular, we see the strongest inverse correlation in TNBC subtypes with either high *TGFBR3* expression (MSL; *P = 5.274e-06*); or low *TGFBR3* expression (basal-like 2; with *P = 5.16e-07* and Luminal AR (LAR); with *P = 1.759e-07)* (Figure 7A-B). The clinical association between *ITGA2* and *TGFBR3* expression is relevant as it further links the impact of the interplay between the TGF-β and integrins pathways in TNBC.

## Discussion

Currently, the functional role of TβRIII is controversial in breast cancer. Some reports suggest a tumor suppressive function of TβRIII [18], while other reports indicate a tumor-promoting role [19,38-40]. Through GE analysis of 587 TNBC patients, we demonstrated that the *TGFBR3* is expressed at a higher level and most consistently in the MSL subtype of TNBC. Furthermore, we have identified MSL cell line models that express high levels *TGFBR3*. To understand better the molecular basis of *TGFBR3* GE we used representative MSL cell lines and a TβRIII loss-of-function approach. The data presented herein support our hypothesis of an oncogenic role for TβRIII in the MSL subtype of TNBC. Biologically, knockdown of TβRIII in TNBC MSL cell lines resulted in decreased motility and invasion, a lack of invasive protrusion in three-dimensional culture *in vitro* and a significant decrease in tumor growth in a xenograft mouse model. The observed migratory and invasive cell line phenotypes were further associated with modulation of the integrin- α2 pathway.

Previously, the loss of TβRIII expression was correlated with progression from a pre-invasive to an invasive state of breast cancer [18]. In addition, restoring expression of TβRIII in a breast cancer cell line led to a decrease of tumor invasiveness *in vitro* and tumor invasion and metastasis *in vivo*[18]. Other studies have demonstrated a frequent loss of TβRIII in human breast cancers [14-17,41]. However, these studies lacked genomic analysis of tumors, thus their difference in conclusions can be due to the difference in their study models. Taking into account the dependency of TGFβ signaling on the context [42] as well as the heterogeneity of breast cancers, especially TNBC [2], we took a more focused approach to study the role of TβRIII in breast cancer progression. Since it has been previously established that TβRIII can modulate TGF-β signaling [6,43-46], it is not surprising that TβRIII has been shown to have both pro- and anti-tumorigenic effects in breast cancer. Our study shows that knockdown of TβRIII in MSL cells appears not to alter the cells’ ability to respond to TGF-β either through the canonical and non-canonical arms of the pathway, or the BMP pathway. Rather, we observed that loss of TβRIII results in a decrease in cell motility and invasion. To further investigate potential mechanisms by which TβRIII regulates these cell functions, we performed gene expression analysis on cells after TβRIII knockdown. We found that the expression of genes involved in integrin signaling and cell-ECM interactions were significantly differentially regulated after TβRIII knockdown.

Previous work has shown that inhibition of integrins can reverse the transformed state of breast cancer cells and that α2β1 integrin can play a role in cancer progression [47]. A more recent study demonstrated that α2β1 integrin acts as a metastasis suppressor in breast cancer, where migratory and invasive abilities of tumor cells are enhanced after loss of α2β1 integrin expression [36]. This supports our finding wherein a decrease in the migratory and invasive phenotype, upon TβRIII knockdown, was linked to increased integrin- α2 expression levels. The precise mechanistic link between TβRIII and integrin- α2 expression levels is unknown. The only other association between integrins and TβRIII was reported in MCF10A breast epithelial cells where TβRIII was shown to regulate integrin- α5 localization [48].

Knockdown of integrin- α2 in TβRIII-KD MSL TNBC cells reverses the loss of motility and invasion that occurs upon TβRIII knockdown alone. One explanation for the observed rescue of migratory and invasive phenotype is through the regulation of ERK phosphorylation possibly mediated by integrin- α2. As shown in Figure 6, upon knockdown of TβRIII we observed a decrease of phospho-ERK simultaneous with an increase in integrin-α2. Furthermore, upon knockdown of integrin-α2 in TβRIII-KD cells we see an increase in phopho-ERK suggesting that integrin-α2 is suppressing ERK activity. This is in agreement with other studies that have shown that integrins can regulate ERK activity [49-51]. In addition, studies have demonstrated that continuous ERK activity can regulate invasion and migration by regulating transcription of genes or directly regulating enzymes necessary for cell movement [52,53]. Therefore, the increase in phospho-ERK seen upon integrin-α2 knockdown could be an explanation for the increase in mobility of our TβRIII-KD cells. Our data show a correlation between TβRIII’s modulation of migration and invasion and the reduction of phospho-ERK levels, possibly mediated by integrin-α2. Further studies will be required to elucidate the precise mechanistic relationship between TβRIII and integrin-α2.

## Conclusions

In summary, our studies using MSL TNBC models demonstrate that TβRIII is an oncogenic driver of migration and invasion *in vitro* as well as tumor growth *in vivo*. Further mechanistic characterization of MSL TNBC would provide insights on how to use this protein and/or signaling pathway as a biomarker or to provide insights to new targets for therapy. Considering the limitations of *in vitro* studies, it is necessary to develop a mouse TβRIII breast cancer model to further elucidate the role of this molecule. Such a model would provide more accurate observations for studying the role of TβRIII in the tumor microenvironment. The results of this study provide mechanistic insight into the role of TβRIII in TNBC and highlight an association between TβRIII and integrin-α2 expression and regulation of cell motility, invasion and tumorigenicity. In addition, this study provides a pre-clinical rationale for consideration of TβRIII as a potential target for further discovery efforts.

## Abbreviations

BL1: basal-like 1; BL2: basal-like 2; BMP: bone morphogenetic protein; DAPI: 4′, 6-diamidino-2-phenylindole; (D)MEM: (Dulbecco’s) modified Eagle’s serum; ECM: extracellular matrix; ER: estrogen receptor; GE: gene expression; HER2: human epidermal growth factor receptor 2; IHC: immunohistochemistry; ITGA2: integrin alpha 2; LAR: luminal androgen receptor; M: mesenchymal; MAtS: magnetic attachable stencils; MSL: mesenchymal stem-like; PBS: phosphate-buffered saline; PR: progesterone receptor; qPCR: quantitative polymerase chain reaction; shRNA: short hairpin RNA; TCGA: The Cancer Genome Atlas; TGFBR3: type III transforming growth factor-beta receptor; TGF-β: transforming growth factor-beta; TGFβ1: transforming growth factor-beta ligand 1; TGFβ2: transforming growth factor-beta ligand 2; TNBC: triple negative breast cancer; TβRI: type I transforming growth factor-beta receptor; TβRII: type II transforming growth factor-beta receptor; TβRIII: type III transforming growth factor-beta receptor; TβRIII-KD: type III transforming growth factor-beta receptor knockdown.

## Competing interests

The authors declare that they have no competing interests.

## Authors’ contributions

BJ was involved in study conception and design, development of methodology, data analyses and interpretation, and writing of the manuscript. JSB was instrumental in assisting with *in silico* data mining and had a significant role in data analysis and interpretation. MWP aided in technical troubleshooting of flow cytometry experiments, as well as with computerized analysis of results. AC performed reporter assays and was involved in data analysis. AEG performed immunoblotting experiments and was involved in data interpretation. WJA provided expertise in magnetic attachable stencils assays as well as help with analysis. BDL provided critical insight on TNBC subtyping and experimental interpretation. AZ, JAP and HLM supervised the study and were primary contributors to study conception, design, and experimental implementation. All authors read and approved the final manuscript.

## Supplementary Material

Additional file 1 Additional information about primer sequences, antibodies and list of GEO registration numbers [2] are referenced in methods section of the manuscript. Click here for file

Additional file 2: Figure S1 Average probe intensities for TGF-β receptors and ligands across 587 TNBC patients. **A-B)** Quantification of *TGFBR1 and TGFBR2* mRNA expression across TNBC tumor subtypes. **C-E)** Quantification of *TGFB1, TGFB2 and TGFB3* mRNA expression. **Figure S2.** Knockdown of TβRIII with two independent shRNA vectors decreases orthotopic tumor volume of SUM159 xenografts. Bars represent mean volume of eight tumors. **Figure S3.** MDA-MB-157 expresses TβRIII after implanted *in vivo* thus does not exhibit significant change in tumor growth. **A)** Bars represent mean tumor volume of 10 tumors. **B)** qRT-PCR comparison of *TGFBR3* expression in MDA-MB-157 cells before implantation and from tumors. **Figure S4.** pSMAD2 and TβRII levels indicate that TGF-β signaling is intact in TβRIII controls and TβRIII-KD MSL lines. Immunoblot analysis. **Figure S5.** TGF-β signaling appears to remain functional in TβRIII-KD MSL cell lines. **A)** Controls and TβRIII-KD MSL cells were examined for CAGA-Luc expression. Bars represents mean of four replicates. **B)** qRT-PCR analysis for *SMAD7* mRNA expression; bars represent the mean of three replicates. **C)** 3TP-lux expression. Bars represent mean of four replicates. **D)** qRT-PCR analysis for *PAI-1* mRNA expression; graph bars represent the mean of three replicates. **Figure S6.** Knockdown of integrin- α2 (α2-KD) in TβRIII-KD MSL cells reverses migratory and invasive TβRIII-KD phenotypes. A) qRT-PCR analysis. **B)** Flow cytometry analysis of α2-KD; arrow pointing to the left shows a shift towards a decrease in integrin-α2 with TβRIII-KD after α2-KD. **C)** Transwell migration assay with α2-KD in TβRIII-KD; bars represents a mean of three replicates. **D)** Transwell invasion assays with inserts pre-coated with matrigel to test for invasion by α2-KD in TβRIII-KD cells. **E)** Immunoblot analysis for phospho-ERK with TβRIII-KD and TβRIII-KD/α2-KD. For all figures, error bars represent SEM, ns = not significant and *P = <0.05, **P = <0.01, ***P = <0.001. Click here for file

Additional file 3: Table S1 Integrin pathway is among significantly changed signaling pathways in SUM159 TβRIII-KD three-dimensional culture system. Genes were considered differentially expressed and included for pathway analysis if they met a cutoff of |log2FC| >0.5 and FDR adjusted *P* value <0.05. Pathway analysis was performed by querying against the C2 Canonical Pathways in the Molecular Signature Database (MSigDB). **Table S2.** Integrin family members in SUM159 cells three-dimensional cultures with TβRIII-KD. Table represents list of integrin family genes from microarray analysis. Genes are ordered based on adjusted *P values* (low to high). *ITGA2* was the top integrin gene with lowest *P value* (*P = 0.003*). Click here for file
