# Supplementary material for: Transforming growth factor beta receptor type III is a tumor promoter in mesenchymal-stem like triple negative breast cancer
Source: Breast Cancer Res. 2014 Jul 1;16(4):R69. doi: 10.1186/bcr3684 (PMC4095685; doi:10.1186/bcr3684)
Supplement: Additional file 1 — Additional information about primer sequences, antibodies and list of GEO registration numbers [2] are referenced in methods section of the manuscript. [file bcr3684-S1.pdf]

File name: **Additional file 1**

Title: **Supplementary Methods**

RNA preparation and quantitative PCR

The following primer sequences (5' to 3') were used:	
**Gene**	**Sequence**
h*TGFBR3* forward	TGGGGTCTCCAGACTGTTTTT
h*TGFBR3* reverse	CTGCTCCATACTCTTTTCGGG
h*PAI-1* forward	GACATCCTGGAACTGCCCTA
h*PAI-1* reverse	GGTCATGTTGCCTTTCCAAGT
h*SMAD7* forward	CCAACTGCAGACTGTCCAGA
h*SMAD7* reverse	CAGGCTCCAGAAGAAGTTGG
h*ITGA2* forward	CCTACAATGTTGGTCTCCCAGA
h*ITGA2* reverse	AGTAACCAGTTGCCTTTTGGATT

Immunoblotting

ANTIBODY (in alphabetical order)	COMPANY	CATALOG #	SPECIES	Concentrations
cleaved Caspase-3	Cell Signaling	9661	rabbit	1:1000
ERK	Cell Signaling	9107	mouse	1:2000
ERK, phospho	Cell Signaling	4370	rabbit	1:2000
PARP	Cell Signaling	9542	rabbit	1:1000
Smad2	Cell Signaling	5339	rabbit	1:1000
Smad2, phospho	Cell Signaling	3101	rabbit	1:1000
TβRII	Santa Cruz	SC400	rabbit	1:4000
TβRIII	SDIX	2703.00.02	rabbit	1:10000

Microarray Gene Expression GEO registration numbers

Breast Cancer GE data sets used to derive TNBC training and validation sets:
GSE-3494 , GSE-7904 , GSE-2109 , GSE-7390 , E-TABM-158 , GSE-2034 , GSE-2990 , GSE-1456 , GSE-22513 , GSE-28821, GSE-28796 , GSE-11121 , GSE-2603 , MDA133 , GSE-5364 , GSE-1561 ,GSE-5327 , GSE-5847 , GSE-12276 , GSE-16446 , GSE-18864 , GSE-19615 , GSE-20194. (*Lehmann BD, Bauer JA, Chen X, Sanders ME, Chakravarthy AB, Shyr Y, Pietenpol JA:* ***Identification of human triple-negative breast cancer subtypes and preclinical models for selection of targeted therapies.*** *The Journal of clinical investigation 2011,* ***121****(7):2750-2767.)*
