# Supplementary material for: Transforming growth factor beta receptor type III is a tumor promoter in mesenchymal-stem like triple negative breast cancer
Source: Breast Cancer Res. 2014 Jul 1;16(4):R69. doi: 10.1186/bcr3684 (PMC4095685; doi:10.1186/bcr3684)
Supplement: Additional file 2: Figure S1 — Average probe intensities for TGF-β receptors and ligands across 587 TNBC patients. A-B) Quantification of TGFBR1 and TGFBR2 mRNA expression across TNBC tumor subtypes. C-E) Quantification of TGFB1, TGFB2 and TGFB3 mRNA expression. Figure S2. Knockdown of TβRIII with two independent shRNA vectors decreases orthotopic tumor volume of SUM159 xenografts. Bars represent mean volume of eight tumors. Figure S3. MDA-MB-157 expresses TβRIII after implanted in vivo thus does not exhibit significant change in tumor growth. A) Bars represent mean tumor volume of 10 tumors. B) qRT-PCR comparison of TGFBR3 expression in MDA-MB-157 cells before implantation and from tumors. Figure S4. pSMAD2 and TβRII levels indicate that TGF-β signaling is intact in TβRIII controls and TβRIII-KD MSL lines. Immunoblot analysis. Figure S5. TGF-β signaling appears to remain functional in TβRIII-KD MSL cell lines. A) Controls and TβRIII-KD MSL cells were examined for CAGA-Luc expression. Bars represents mean of four replicates. B) qRT-PCR analysis for SMAD7 mRNA expression; bars represent the mean of three replicates. C) 3TP-lux expression. Bars represent mean of four replicates. D) qRT-PCR analysis for PAI-1 mRNA expression; graph bars represent the mean of three replicates. Figure S6. Knockdown of integrin- α2 (α2-KD) in TβRIII-KD MSL cells reverses migratory and invasive TβRIII-KD phenotypes. A) qRT-PCR analysis. B) Flow cytometry analysis of α2-KD; arrow pointing to the left shows a shift towards a decrease in integrin-α2 with TβRIII-KD after α2-KD. C) Transwell migration assay with α2-KD in TβRIII-KD; bars represents a mean of three replicates. D) Transwell invasion assays with inserts pre-coated with matrigel to test for invasion by α2-KD in TβRIII-KD cells. E) Immunoblot analysis for phospho-ERK with TβRIII-KD and TβRIII-KD/α2-KD. For all figures, error bars represent SEM, ns = not significant and *P = <0.05, **P = <0.01, ***P = <0.001. [file bcr3684-S2.pdf]

Figure S1.

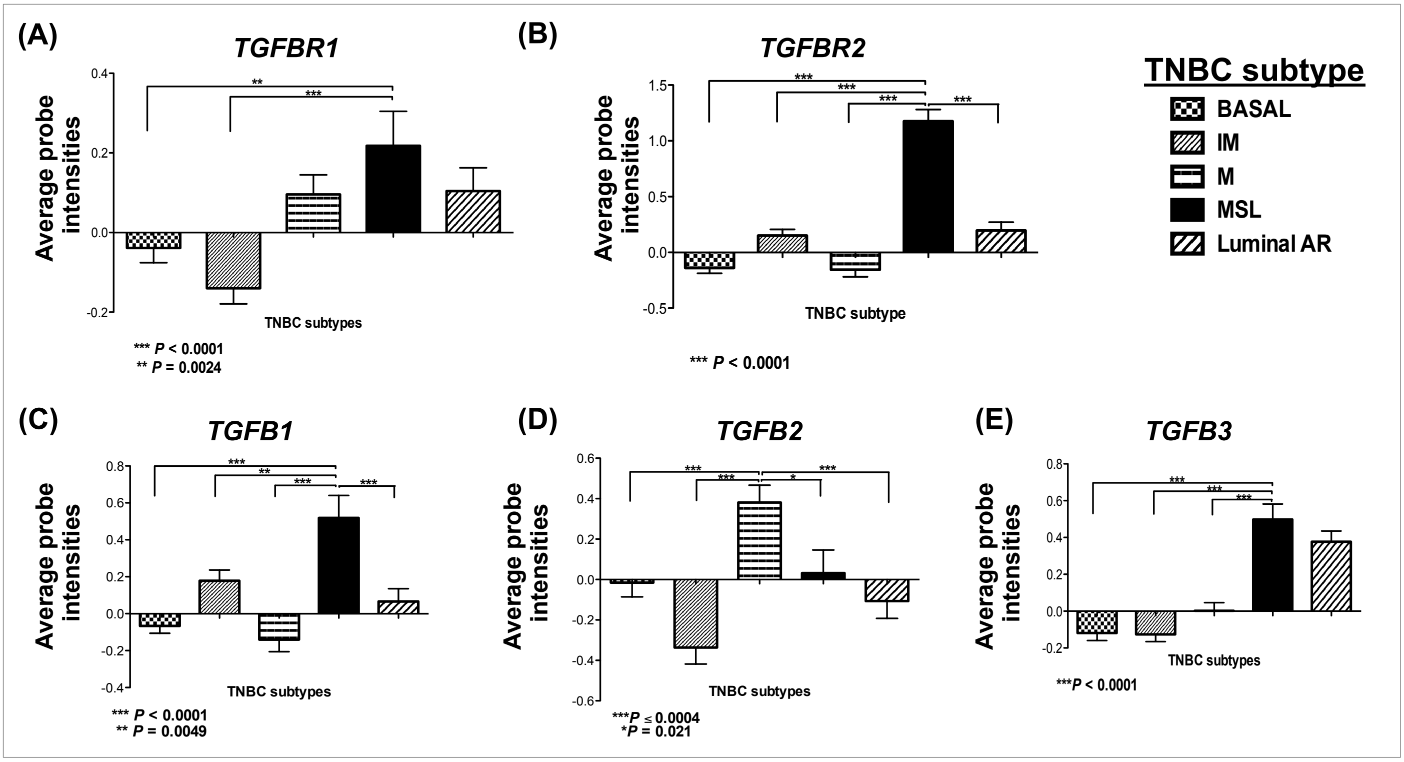
(A)
TGFBR1
Average probe intensities
TNBC subtypes
*** P < 0.0001
** P = 0.0024
(B)
TGFBR2
Average probe intensities
TNBC subtype
*** P < 0.0001
TNBC subtype
BASAL
IM
M
MSL
Luminal AR
(C)
TGFB1
Average probe intensities
TNBC subtypes
*** P < 0.0001
** P = 0.0049
(D)
TGFB2
Average probe intensities
TNBC subtypes
***P ≤ 0.0004
*P = 0.021
(E)
TGFB3
Average probe intensities
TNBC subtypes
***P < 0.0001

Figure S2.

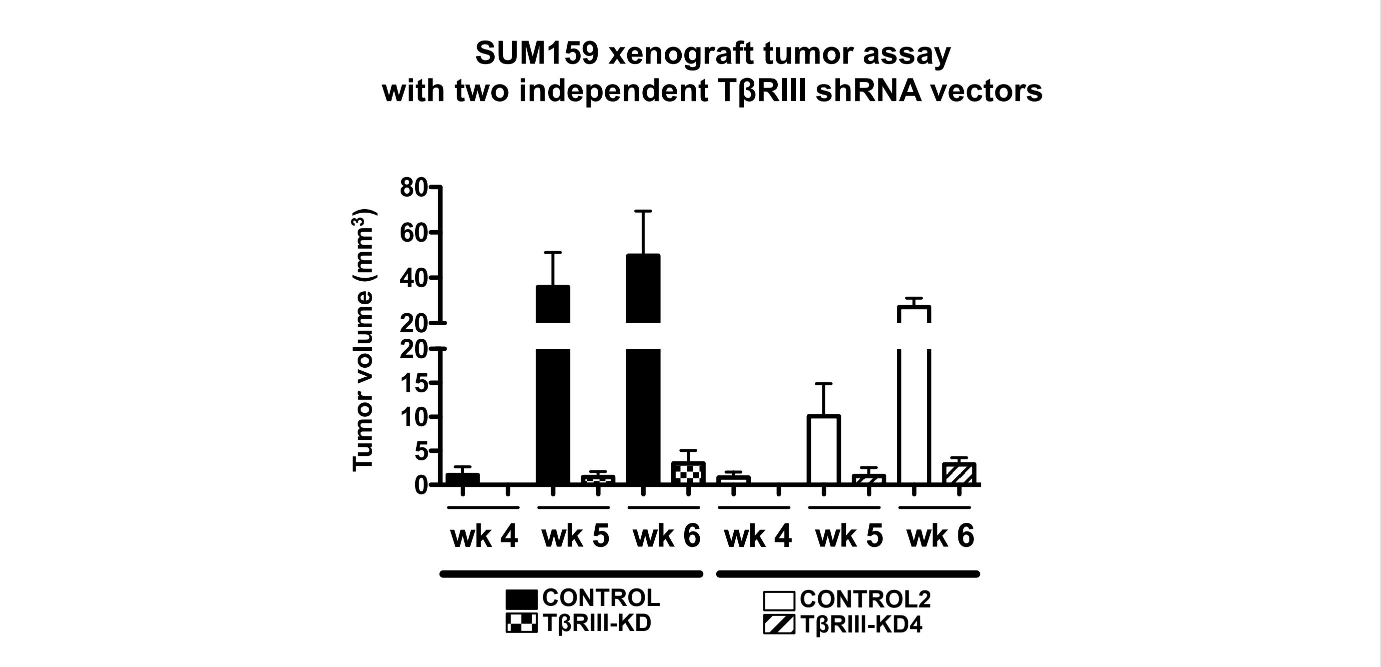

SUM159 xenograft tumor assay
with two independent TβRIII shRNA vectors
Tumor volume (mm3)
80
60
40
20
20
15
10
5
0
wk 4
wk 5
wk 6
wk 4
wk 5
wk 6
CONTROL
TβRIII-KD
CONTROL2
TβRIII-KD4

Figure S3.

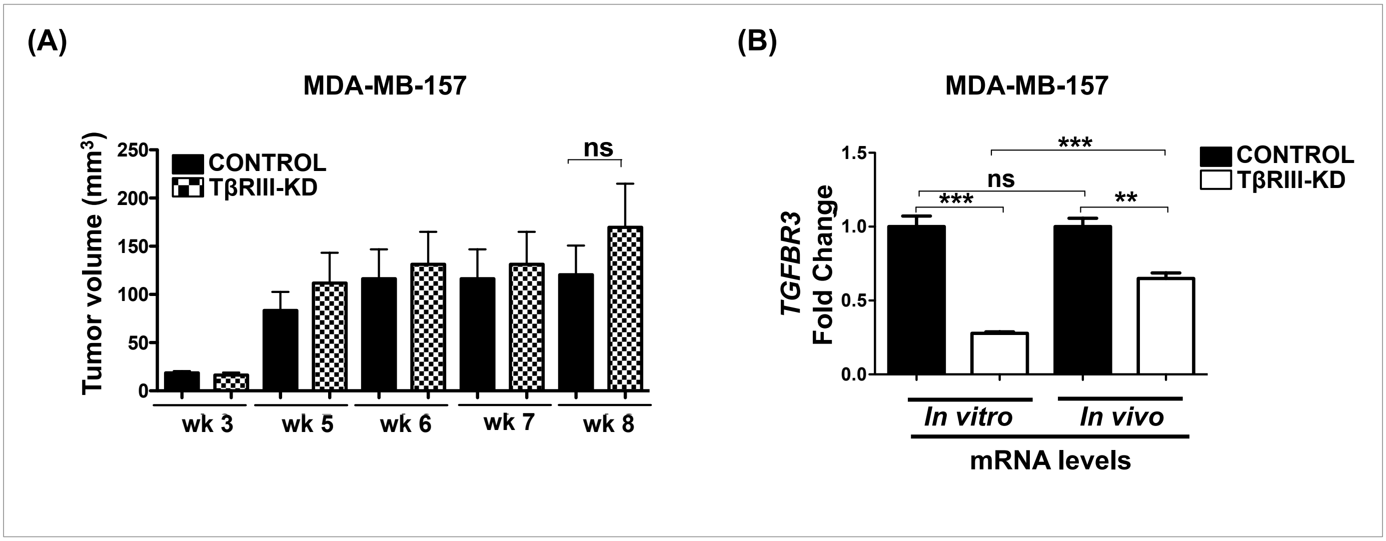

(A)
MDA-MB-157
Tumor volume (mm3)
250
200
150
100
50
0
CONTROL
TβRIII-KD
ns
wk 3
wk 5
wk 6
wk 7
wk 8
(B)
MDA-MB-157
TGFBR3 Fold Change
1.5
1.0
0.5
0.0
CONTROL
TβRIII-KD
***
ns
***
**
In vitro
In vivo
mRNA levels

Figure S4.

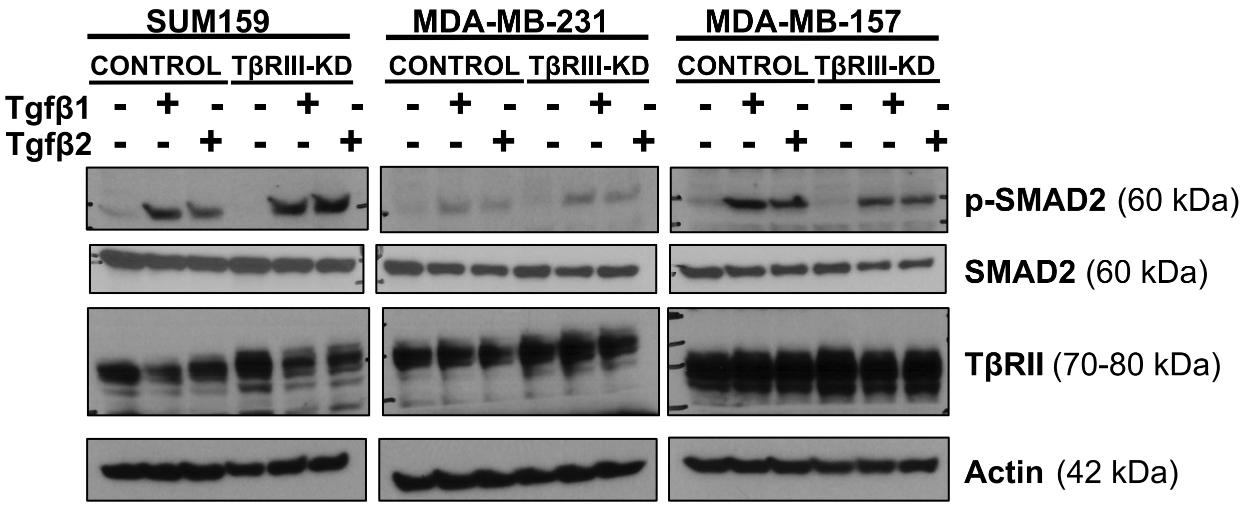
SUM159
MDA-MB-231
MDA-MB-157
CONTROL
TβRIII-KD
Tgfβ1
Tgfβ2
p-SMAD2 (60 kDa)
SMAD2 (60 kDa)
TβRII (70-80 kDa)
Actin (42 kDa)

Figure S5.

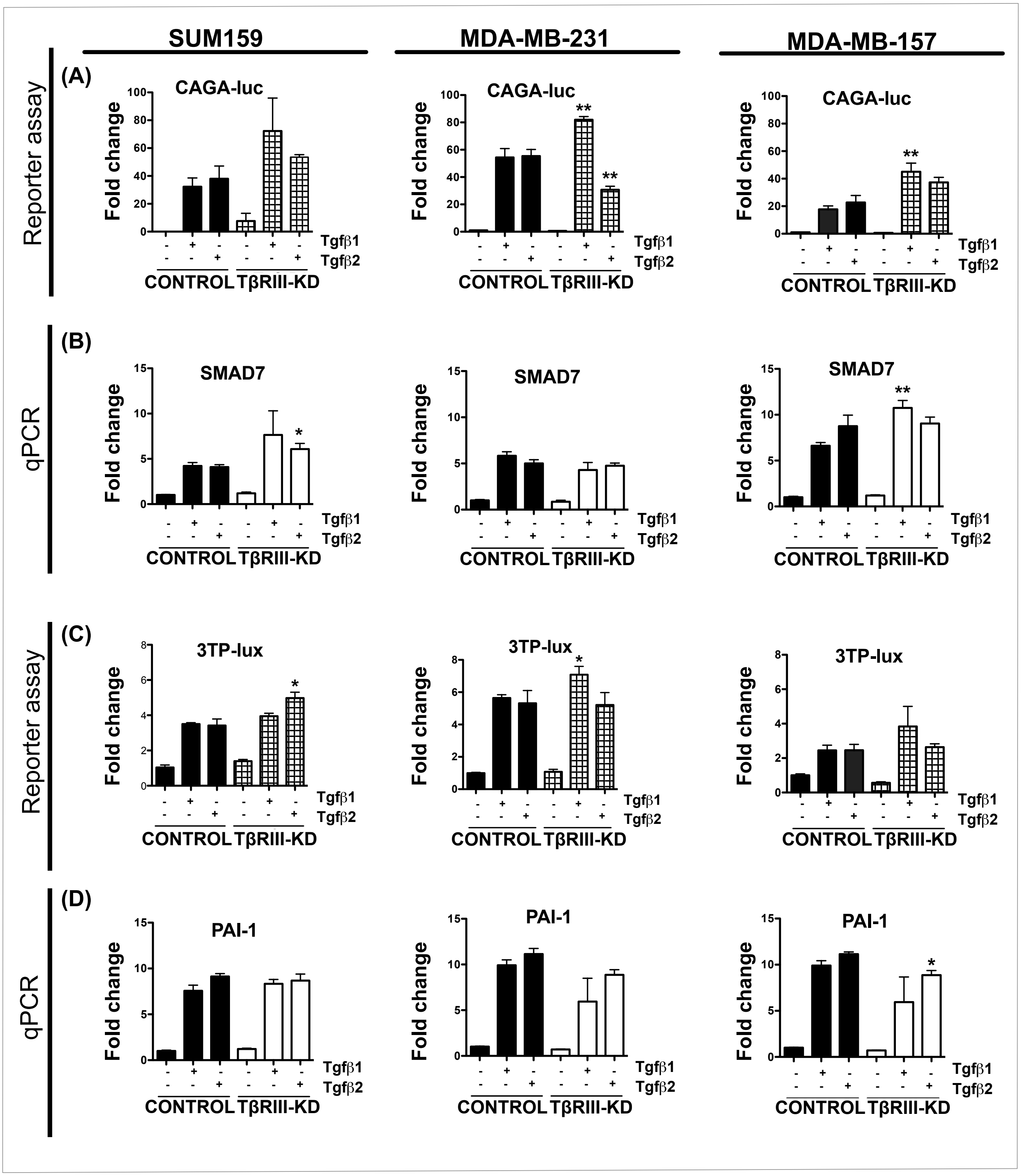
SUM159
MDA-MB-231
MDA-MB-157
Reporter assay
(A)
CAGA-luc
Fold change
Tgfβ1
Tgfβ2
CONTROL
TβRIII-KD
**
qPCR
(B)
SMAD7
*
(C)
3TP-lux
(D)
PAI-1

Figure S6.

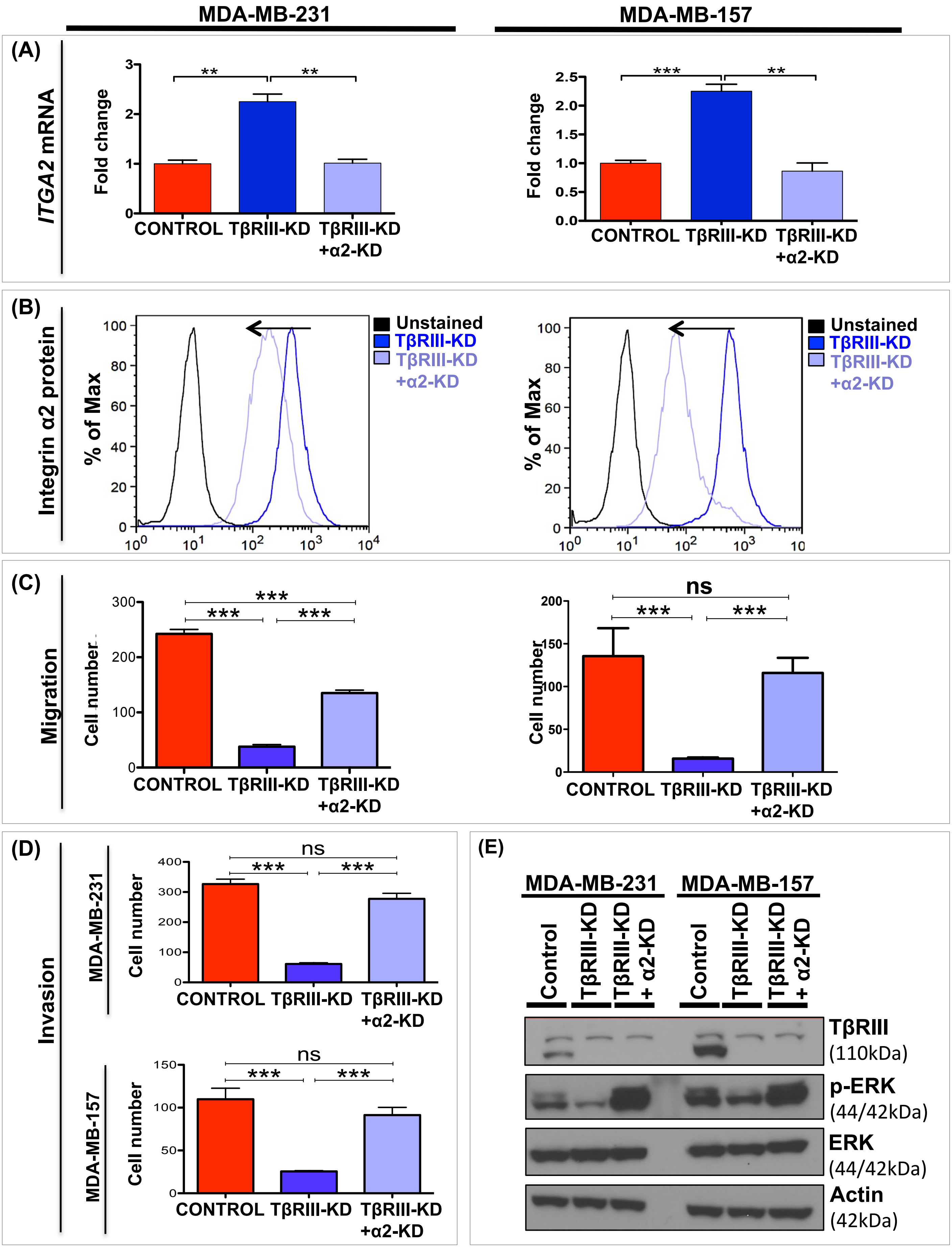

MDA-MB-231
MDA-MB-157
(A)
ITGA2 mRNA
Fold change
CONTROL
TβRIII-KD
TβRIII-KD +α2-KD
(B)
Integrin α2 protein
% of Max
Unstained
TβRIII-KD
TβRIII-KD +α2-KD
(C)
Migration
Cell number
ns
(D)
Invasion
MDA-MB-231
MDA-MB-157
Cell number
ns
(E)
MDA-MB-231
MDA-MB-157
Control
TβRIII-KD
TβRIII-KD + α2-KD
TβRIII (110kDa)
p-ERK (44/42kDa)
ERK (44/42kDa)
Actin (42kDa)
