# Supplementary material for: Transforming growth factor beta receptor type III is a tumor promoter in mesenchymal-stem like triple negative breast cancer
Source: Breast Cancer Res. 2014 Jul 1;16(4):R69. doi: 10.1186/bcr3684 (PMC4095685; doi:10.1186/bcr3684)
Supplement: Additional file 3: Table S1 — Integrin pathway is among significantly changed signaling pathways in SUM159 TβRIII-KD three-dimensional culture system. Genes were considered differentially expressed and included for pathway analysis if they met a cutoff of |log2FC| >0.5 and FDR adjusted P value <0.05. Pathway analysis was performed by querying against the C2 Canonical Pathways in the Molecular Signature Database (MSigDB). Table S2. Integrin family members in SUM159 cells three-dimensional cultures with TβRIII-KD. Table represents list of integrin family genes from microarray analysis. Genes are ordered based on adjusted P values (low to high). ITGA2 was the top integrin gene with lowest P value (P = 0.003). [file bcr3684-S3.pdf]

Table S1. Integrin pathway is among significantly changed signaling pathways in SUM159 TβRIII-KD three-dimensional culture system

Gene Set Name	# Genes in Gene Set (K)	# Genes in Overlap (k)	p-value
REACTOME_CELL_JUNCTION_ORGANIZATION	78	8	7.67E-07
REACTOME_CELL_CELL_COMMUNICATION	120	9	2.24E-06
PID_INTEGRIN1_PATHWAY	66	7	3.03E-06
KEGG_CELL_ADHESION_MOLECULES_CAMS	134	8	4.31E-05
PID_INTEGRIN3_PATHWAY	43	5	5.20E-05
KEGG_FOCAL_ADHESION	201	9	1.35E-04
REACTOME_CELL_CELL_JUNCTION_ORGANIZATION	56	5	1.87E-04
PID_AVB3_INTEGRIN_PATHWAY	75	5	7.29E-04
REACTOME_INTEGRIN_CELL_SURFACE_INTERACTIONS	79	5	9.23E-04
KEGG_ECM_RECEPTOR_INTERACTION	84	5	1.22E-03
REACTOME_EXTRACELLULAR_MATRIX_ORGANIZATION	87	5	1.42E-03
REACTOME_ADHERENS_JUNCTIONS_INTERACTIONS	27	3	2.05E-03

Table S2. Integrin family members in SUM159 cells three-dimensional cultures with TβRIII-KD

Gene symbol	Adj. P value
ITGA2	0.00310994
ITGB2	0.00858093
ITGA6	0.07342169
ITGB5	0.18804443
ITGB1	0.2823499
ITGA8	0.35612028
ITGA3	0.39006295
ITGA4	0.49020532
ITGB7	0.50179171
ITGA10	0.50579062
ITGB4	0.56483922
ITGA11	0.65315048
ITGB3	0.69170401
ITGB6	0.70117658
ITGA7	0.74009154
ITGAV	0.78396341
ITGA5	0.78412514
ITGB8	0.94109833
ITGA9	0.95105451
